# Relative contribution of groundwater to plant transpiration estimated with stable isotopes

**DOI:** 10.1038/s41598-017-09643-x

**Published:** 2017-09-05

**Authors:** Adrià Barbeta, Josep Peñuelas

**Affiliations:** 1 ISPA, Bordeaux Science Agro, INRA, 33140 Villenave d’Ornon, France; 2 CSIC, Global Ecology Unit CREAF-CSIC-UAB, E-08193 Bellaterra (Catalonia), Spain; 3 CREAF, E-08193 Cerdanyola del Vallès (Catalonia), Spain

## Abstract

Water stored underground in the saturated and subsurface zones below the soil are important sources of water for plants in water-limited ecosystems. The presence of deep-rooted plants worldwide, however, suggests that the use of groundwater is not restricted to arid and seasonally dry ecosystems. We compiled the available data (71 species) on the relative contribution of groundwater to plant water estimated using stable isotopes and mixing models, which provided information about relative groundwater use, and analyzed their variation across different climates, seasons, plant types, edaphic conditions, and landscape positions. Plant use of groundwater was more likely at sites with a pronounced dry season, and represented on average 49 per cent of transpired water in dry seasons and 28 per cent in wet seasons. The relative contribution of groundwater to plant-water uptake was higher on rocky substrates (saprolite, fractured bedrock), which had reduced groundwater uptake when this source was deep belowground. In addition, we found that the connectivity between groundwater pools and plant water may be quantitatively larger and more widespread than reported by recent global estimations based on isotopic averaged values. Earth System Models should account for the feedbacks between transpiration and groundwater recharge.

## Introduction

Water pools stored underground in the saturated zone (i.e. groundwater^1^) are accessed by the deep roots of plants. Aquifers influence many terrestrial ecosystems^2^, and many other ecosystems depend partly on water-saturated fractures in bedrock and on water from unsaturated vadose zones below the soil^3–5^. Groundwater is ultimately transpired to the atmosphere through foliar stomata, sometimes increasing atmospheric humidity and modifying regional climate^6^. The interactions between the performance of the vegetation and the level of the water table at the catchment scale can modify the contribution of groundwater to streamflow^7^. The fluctuations of water-table levels or the quantity of water trapped within fractured bedrock have different spatiotemporal dynamics than upper soil moisture but may have a comparable importance for ecosystem water budgets and plant growth^8^. Indeed, the contribution of groundwater to plant transpiration may be critical in ecosystems with transient or chronic water deficits^9^. But studies of plant-water source use, however, have not measured routinely the uptake of groundwater. This has been attributed to technological and economic limitations and to the widespread assumption that deep roots are a secondary component of a plant root system^10^. Since the depth to groundwater is shallow enough to influence vegetation over notable portions of land area^2, 11^, attempts to integrate groundwater into Earth System Models have increased in recent years^11–13^. In order to improve the representation of groundwater-surface interactions in models, a quantification of the relative contribution of groundwater to transpiration and its variability across environmental gradients is required.

Water is generally taken up by roots (with important exceptions^14^), so root structure and function should play a central role in research of plant-water relations. In the last two decades several studies have compiled and synthesized global-scale data on the maximum rooting depth^9, 15^ and the root distribution along soil profiles and across climates and plant types^16^ and have modeled the probability of deep rooting globally^17^. Arid and seasonally dry ecosystems contain the deepest root systems^9^, and some species grow roots to depths of more than 4 m, even in temperate and tropical ecosystems^17^. Plants around the world (perhaps with the exception of boreal and polar regions) thus grow deep roots. All likely use deep water reserves to some extent, a capacity that will confer a competitive advantage in regions where an increase in drought frequency, intensity, and/or duration is projected^18^. Deep rooting also facilitates plant coexistence by segregating their hydraulic niches, even in ecosystems where water is not limiting^19, 20^. The existing literature reporting the use of groundwater pools by roots is extensive, but we still lack a quantitative assessment of the relative contribution of those pools at a broad geographical scale.

The activity of deep roots can be traced with the stable isotopes of water (δ^18^O and δ^2^H). These isotopes are able to successfully identify the source of transpired water by a simultaneous comparison of the isotopic compositions of both xylem and source water^21^. Sources from which plants take up water (soil water at different depths, fog, dew, or groundwater) usually have different isotopic compositions because of evaporative fractionation^22^ and the rainout effect^23^. The uptake of water by roots generally involves little fractionation (but there are exceptions^24^), so the source of xylem water can often be identified from among pools with contrasting isotopic signatures^21^.

A recent multi-site analysis of the isotopic compositions of precipitation, soil water, plant xylem water, groundwater, and stream water suggested the widespread ecohydrological separation of groundwater (not used by plants) and soil water (always used by plants)^25^. The “two water worlds hypothesis”^26^ was proposed following the isotopic characterization of soil and groundwater water pools in two contrasting sites^23, 27^. Based on a two-pool model the authors infer that plants appear to take up water preferentially from an isotopically distinct water pool (soil) that is clearly different from the water that is delivered to aquifers and streams^23^. Whether the compartmentalization of below-ground water pools is (i) physical: plants take up water “tightly” bound in soil micropores, and mobile water in macropores contributes to the recharge of groundwater and stream water or (ii) temporal: groundwater is recharged at a different time than plant-water uptake^28^ is something that remains unclear. Subsequent studies did not find any evidence to attribute isotopic differences between plant-accessed water and groundwater solely to soil water mobility^29^ or to the temporal segregation of groundwater recharge and plant transpiration^30^. Furthermore, the ubiquity of the ecohydrologic compartmentalization does have notable exceptions^31, 32^ that were not part of the abovementioned meta-analysis. A recent review presented an alternative explanation for the isotopic differences between plant transpired water (and topsoil water) and groundwater^33^. According to this review, precipitation events result in the mixing of non-fractionated water with isotopically enriched topsoil water. This mixed water percolates to increasing depths but the relatively small volume of enriched water produces a dampening of the evaporated signal with depth. As a result, recharge water showing a non-fractionated signal does not imply that vegetation uses a different pool of pore water^33^, which challenges the hypothetical compartmentalization or hydrologic disconnection between recharge/drainage water and plant-accessed water^28^. At this point, a better understanding of the connections between plant transpiration and groundwater pools would help elucidate some of the questions arising from these recent findings.

In the face of current climate change, a more comprehensive understanding of the relationship between groundwater and other subsurface water pools below the soil (saprolite water, bedrock-related water) and vegetation is therefore crucial. We have thus quantified the relative contributions of groundwater pools to plant transpiration by collecting data from studies (Table S1) that applied stable-isotope techniques and isotope mixing models to calculate water-source contributions. We targeted studies evaluating the contributions of groundwater and sampled the broadest geographical scale possible. We used a quantitative synthesis to (i) compare groundwater use between contrasting climatic and edaphic conditions, plant functional types and landscape positions, (ii) assess the relative contributions of these factors to the variation in groundwater use, (iii) define the extent of the hydrological connectivity between groundwater and plant transpiration and (iv) quantify the potential effects of isotopic methodologies.

## Results

**Figure 1:**
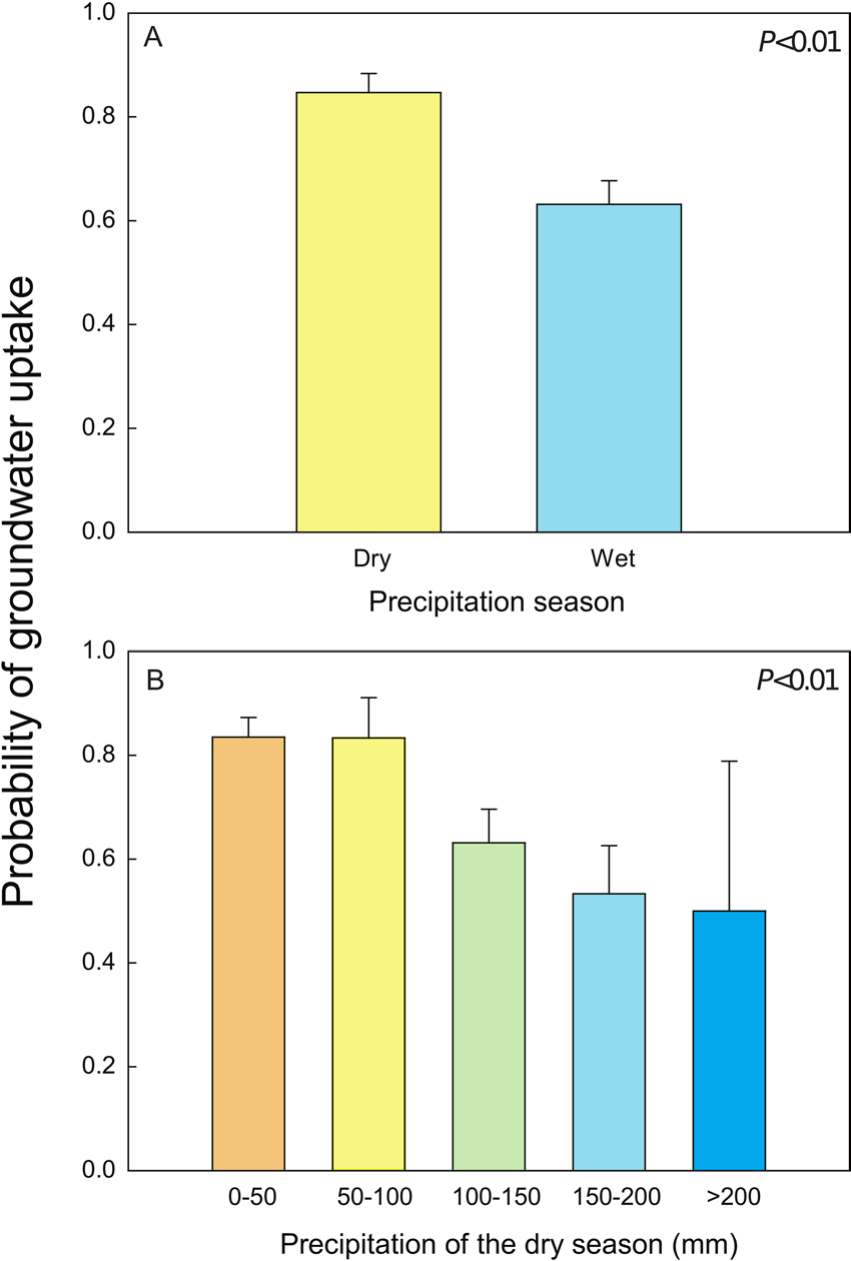
Probability of groundwater uptake. The probability of groundwater uptake in (**A**) dry and wet seasons and (**B**) as a function of the amount of precipitation during the dry seasons. Error bars are the standard errors of the means, and P was calculated with the GLMMs from the *MCMCglmm* package in R.

The probability of at least some plant groundwater uptake (estimated contributions of groundwater to plant water higher than 10%) was 84.2 ± 3.7% in dry seasons and 64.1 ± 4.4% in wet seasons. Accordingly, the probability of groundwater uptake was mainly determined by the precipitation season (dry or wet season) and the mean precipitation amount during the dry season. That is, groundwater is therefore more likely to be taken up in dry seasons (Fig. 1A) and at the sites with lower precipitation during those dry seasons (Fig. 1B). Climate seasonality was thus the main factor determining the probability of groundwater uptake, whereas landscape position or edaphic conditions did not generally have significant effects. We also found trees more likely to use groundwater than shrubs (Table S2).

**Figure 2:**
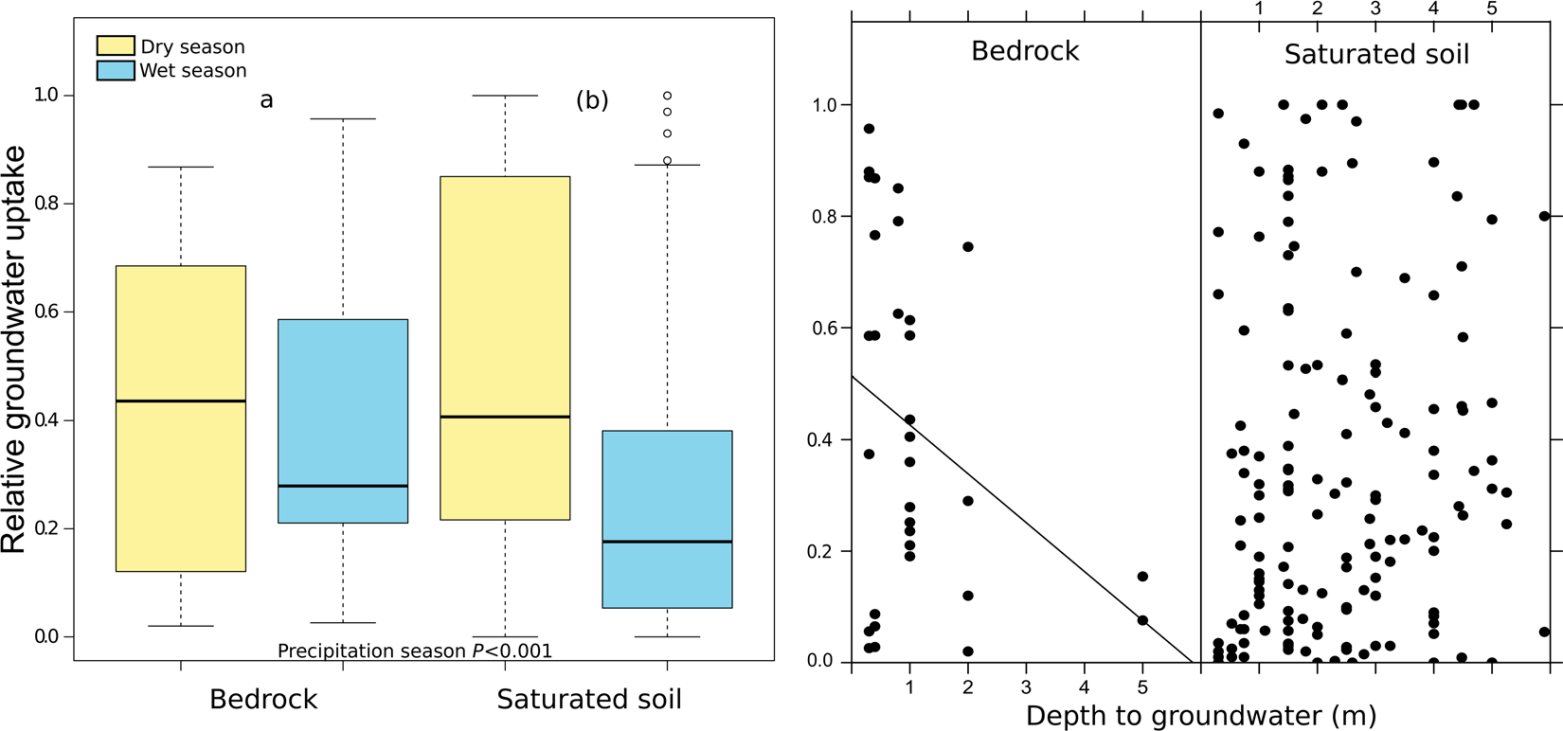
Relative groundwater uptake and edaphic conditions. (**A**) Boxplot of the relative groundwater uptake in dry and wet seasons and different edaphic conditions. Box size represents the interquartile range, the black line is the median, the whiskers indicate variability outside the upper and lower quartiles, and individual points are outliers. A different letter in parenthesis indicates a marginally significant difference (*P* < 0.1) between edaphic conditions. (**B**) The linear effect of the depth to groundwater (log-transformed) on the relative groundwater uptake for the two edaphic conditions.

**Figure 3:**
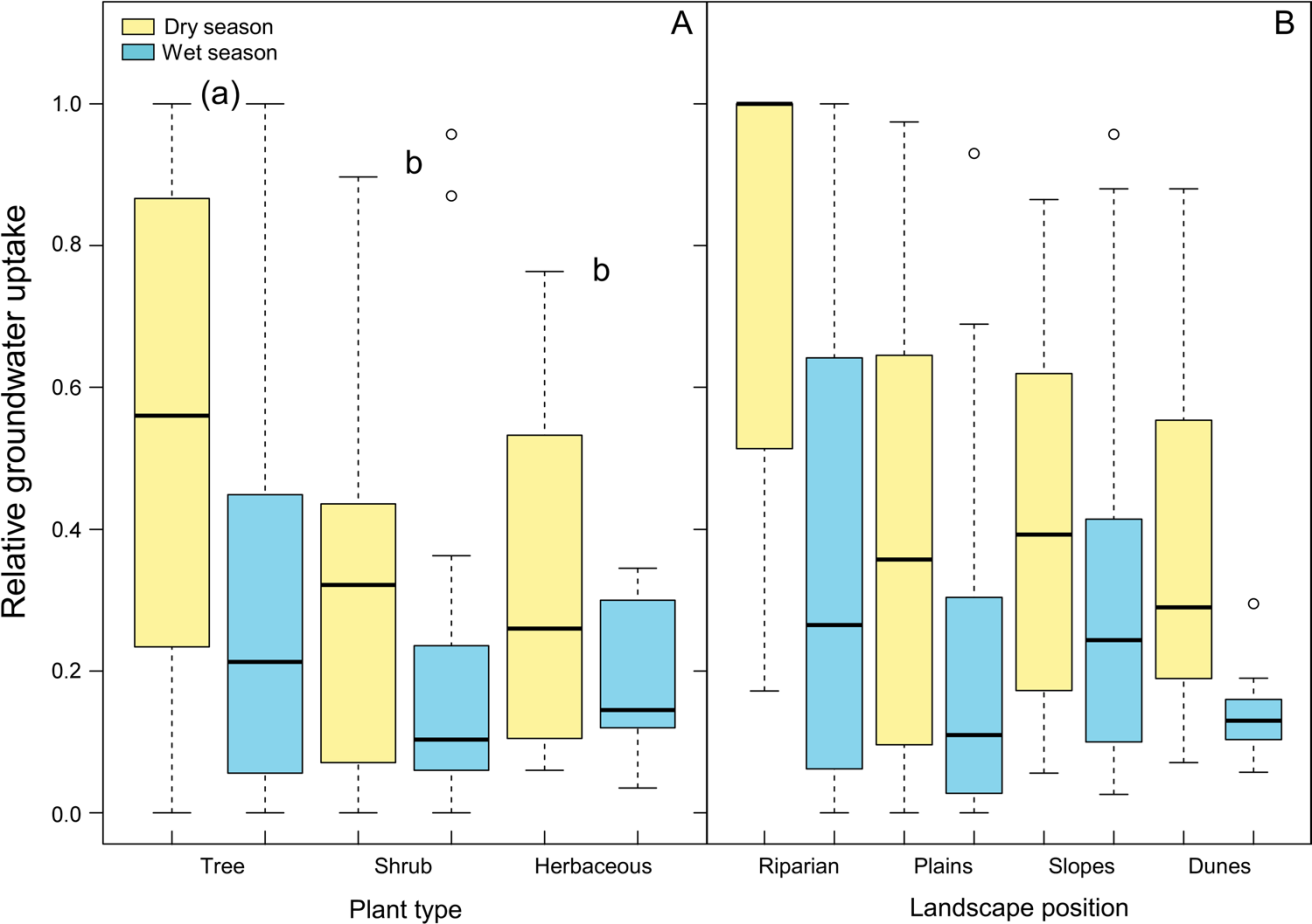
Relative groundwater uptake by plant type and landscape position. Boxplots of the relative groundwater uptake in dry and wet seasons for different (**A**) plant types and (**B**) landscape position. Box size represents the interquartile range, the black line is the median, the whiskers indicate variability outside the upper and lower quartiles, and individual points are outliers. A different letter in parenthesis indicates a marginally significant difference (*P* < 0.1) between groups.

The relative contribution in cases with substantial groundwater uptake (N = 155) was significantly affected by season, edaphic conditions, and plant type (Table S2). In those cases, the relative contribution of groundwater uptake was higher in dry seasons (55.98% vs. 31.15%, *P* < 0.001). The relative contribution to plant-water uptake was higher at sites where the groundwater was in saturated fractured bedrock than at sites where groundwater was in saturated soil layers (41.8% vs. 36.4%, *P* < 0.001) (Fig. 2A). The deeper the groundwater pools were, the lower their relative contribution was, but only in those cases where the groundwater was within the bedrock (Fig. 2B). Plant functional type was a marginally significant factor determining the relative contribution of groundwater; tree and shrub species took up more groundwater than herbaceous species (Fig. 3A). The average relative contribution of groundwater varied with landscape position (Fig. 3B). Plants growing in riparian ecosystems have the highest contributions of groundwater in dry seasons, with the median use near 100%. Landscape position, however, was not a significant factor and was highly variable within each of the defined levels (riparian, plains, slopes, and dunes) (Fig. 3B).

**Figure 4:**
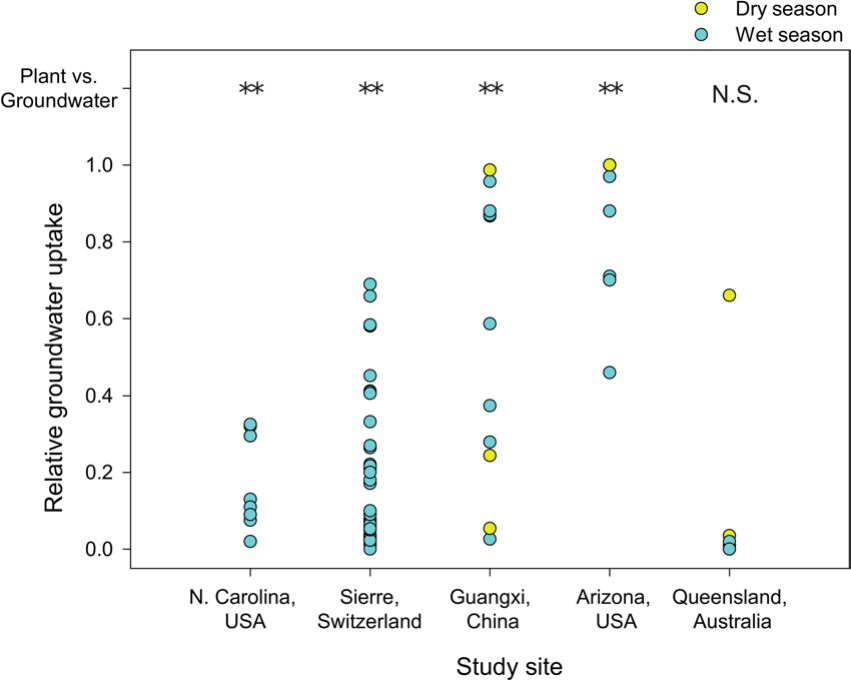
Evaluation of the hydrological connectivity between plant water and groundwater. Relative groundwater uptake of individual cases of studies reviewed by Evaristo and colleagues (Evaristo *et al*.^25^) and the present study. Yellow circles indicate contributions of groundwater to plant transpiration in dry seasons, and blue circles are wet seasons. Each point belongs to a single species and precipitation season. Above each study, asterisks (**) denote significant statistical differences between the isotopic composition of plant water and groundwater, as calculated in ref. (Evaristo *et al*.^25^). N.S. indicates that significant differences were not found by ref. 27. Notice the substantial number of cases with relative groundwater uptake higher than 10% even in the four sites where Evaristo and colleagues (27) found significant differences between the isotopic composition of plant water and groundwater.

Our dataset contained five studies that were also included in the multi-site analysis of Evaristo and colleagues^25^. This study stated that there is a ubiquitous separation of groundwater and plant xylem water, which only came from the soil. In four out of those five studies, they reported that groundwater and plant-accessed water feed from two different water pools owing to statistical differences in their isotopic compositions. In Fig. 4, we show the estimations made by the authors of the studies compiled by both Evaristo and colleagues and the present study. Data was split by species, showing instead significant contributions of groundwater to plant water uptake, in many cases constituting the most important plant water source. In the only study in which groundwater and plant water were not found to be isotopically distinct by the Evaristo and colleagues approach^25^, only one case showed significant groundwater uptake.

The relative uptake of groundwater by plants for all data analyzed had means of 48.8 ± 3.4% in dry seasons and 27.8 ± 2.6% in wet seasons (mean and standard error of the mean). These estimates, however, are susceptible to the sampling design and application of the isotope mixing model. The number of potential sources considered in the models particularly affected the estimates, because groundwater can isotopically resemble other belowground sources within unsaturated soil. The General Linear Mixed Model (GLMM) indicated that the number of potential plant water sources measured and included in the model significantly reduced the estimation of relative groundwater use (P = 0.019). Indeed, the median estimation of groundwater use when only two water sources were considered was higher (Fig. 4) compared to when three or four water sources were considered. Estimations of groundwater use, however, were higher in studies considering five or more potential plant water sources than in those considering three or four. The median estimation of the relative plant groundwater uptake can therefore be affected by the sampling design of each study. The estimation in this study ranged from 62.8% (two sources) to 37.9% (four sources) for the dry seasons and from 35.9% (two sources) to 10.4% (four sources) for the wet seasons (Fig. 4). The number of isotopes used for the estimation of relative groundwater uptake affected the precision of the measure but likely had no effect on our overall results. The range of feasible solutions using the IsoSource mixing model (P < 0.05) was significantly narrower in studies with both water isotopes than in those using only one of the isotopes (Fig. S1A). The estimated medians or means (P = 0.83), however, did not differ between a dual- or single-isotope analyses (Fig. S1B).

## Discussion

### General Findings

The average relative contribution of groundwater to plant water uptake was 49% in dry seasons and 28% in wet seasons. Groundwater uptake was significant in 84.2% of the cases during dry seasons and 64.1% of the cases in wet seasons. The cases in the present analysis therefore indicated that plant groundwater use was certainly widespread, occurring at least to some extent in 90.1% of the species studied (N = 71). The ecosystems selected by the studies in the analysis, however, were most likely to have occurred at sites with groundwater present and where this water source was hypothesized to have an influence on vegetation. We thus believe that the relative quantities and probabilities of groundwater uptake are basically representative of groundwater-dependent ecosystems. The analysis, though, included both true phreatophytes (e.g. *Salix gooddingii*) and other species with broader distributions along topographical gradients of water availability (e.g. *Pinus sylvestris*). Not all plant water source studies consider groundwater as a potential source. If we had included all those studies, the estimation of groundwater uptake will be certainly lower^34^. However, not considering all potential water sources can produce spurious source water estimations by mixing-models^35^. Therefore, assuming no groundwater uptake in certain sites solely because groundwater was not sampled is at the risk of underestimating its contribution. In turn, our estimations of groundwater uptake are not as general, but more reliable for the ecosystems included in this synthesis.

**Figure 5:**
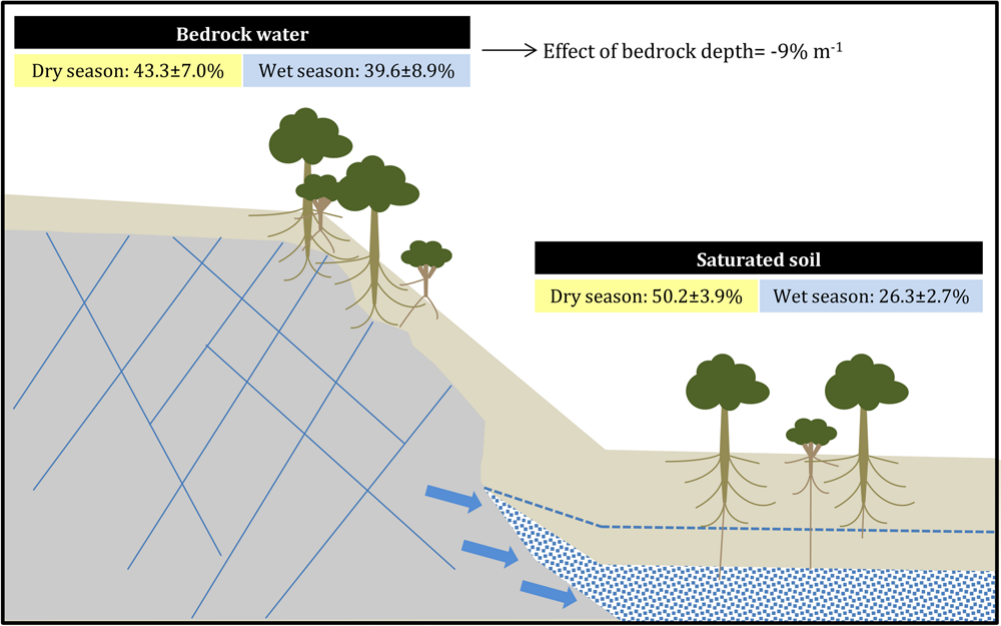
Schematic representation of the contribution of belowground water pools to plant water. This study reviewed studies quantifying the contribution of bedrock water (left of the picture) and saturated soil layers (right of the picture) to plant water. The mean contribution of groundwater and the standard error of the mean for each type of groundwater and precipitation season are shown in the boxes. In dark grey, the fractured and/or weathered bedrock that plants tap. The light grey represents the soil, and the blue dotted area in the plain is the saturated soil layers. Dashed line represents the water table level in wet seasons. The effect of the depth to groundwater is based on the GLMM and was only significant for bedrock water.

Our results are consistent with the common occurrence of deep roots in woody and herbaceous species in all biomes of the world^9^. An estimated 95% of the rooting distribution along soil profiles ranged between 0.3 m in tundra to 1.7 m in Mediterranean woody ecosystems^16^. In the studies summarized here, groundwater was extracted by roots from a mean depth of 3.05 ± 0.30 m. Groundwater pools and deep root biomass therefore importantly contribute to total plant transpiration^9^, impacting evapotranspiration and should thus be properly incorporated into Earth System Models (ESM). In recent years, ESMs have attempted to represent groundwater dynamics through diverse approaches, and the key challenge remains to account for the spatial variability in water table depth^12^. In this regard, we show here that groundwater is not only important in sites with shallow water tables, but also in sites with plants growing on shallow and rocky soils (Figs 2 and 5). Similarly, we found a significant contribution of groundwater to transpiration in all landscape positions (Fig. 3), which suggests that it will be necessary to expand the representation of groundwater dynamics in ESM across the landscape, including hillslopes. The absence of correlation between the depth to groundwater and the relative contribution of groundwater to transpiration when this water source is found in saturated soil layers (Fig. 2) may illustrate that rooting depth and root function (e.g., uptake^36^) varies spatially as a function of the depth to groundwater. Unless there is a physical impediment such as hard bedrock, rooting depth will expand down to the water table and for that matter where water resources are more available (many plants can be opportunistic with their resource use) (Fig. 5). This is in agreement with the idea that rooting depth is determined by gradients in the water table depth^11^ and where resources are most available.

Climate was the major factor determining the probability of groundwater uptake. And, as expected because climatic features, especially site water balance are key drivers driving the distribution of deep roots^9^ it is not surprising to see that this is a global generality. Groundwater uptake was significantly more likely in dry seasons (Fig. 1A), consistent with the prevalence of marked seasonality of water uptake in many species, as first reported for *Eucalyptus* and *Banksia* trees^22^ as well as for *Quercus* in Spain^37^ and California (T. Dawson, pers. comm.). The probability of groundwater uptake was even higher at sites with a more extreme dry season (effect of precipitation of the driest quarter, Fig. 1B). Plant groundwater uptake was more probable when surface soils were more likely to be dry due to the lack of precipitation, as expected.

Climate, edaphic characteristics, and plant type explained most of the variance in the relative contribution of groundwater uptake. Climatic seasonality was the most important factor, because plants are not only more likely to extract groundwater in dry seasons but also use higher relative quantities (Table S3). This seasonal pattern is common and has been observed in arid, temperate, and tropical ecosystems^11, 37–40^. Thus, our data set supported the ubiquity of the seasonality of plant groundwater use. Groundwater is therefore a water source that alleviates water stress during dry seasons when surface soil moisture is depleted, and its proportional use decreases in wet seasons. The capacity of the surface soil to retain moisture throughout the dry season will depend on its depth and edaphic characteristics. Plants can grow larger and resist droughts better in soils with well-developed top layers than in rocky soils^41^. Groundwater may indeed a critical water source at sites where roots can and do penetrate deep into bedrock fractures to seek pockets of moisture^3, 42^. In fact, the groundwater in our study taken up directly from these rock fractures contributed more to plant-water uptake than groundwater found deep soil saturated layers, noticeably during dry seasons (Fig. 2A). If groundwater is associated with bedrock, the contribution to plant water is negatively correlated with the depth to the bedrock (Fig. 2B). Therefore, plants growing in shallow surface soils strongly depend on water trapped in rock fractures. In contrast, the relative use of groundwater by plants growing on deep soils with a saturated non-rocky layer, has not been affected by the depth to groundwater (Fig. 2B). The additional water supply provided by groundwater pools was also critically determined by plant functional type. Trees were more likely to use groundwater than shrubs (Table S2), and tended to use higher relative amounts than herbaceous species (Fig. 3A, Table S3). Trees have deeper roots than other plant types^16^, and the size of trees has been associated with higher groundwater access^43^. A tree growing on shallow and rocky soil under a climate with a pronounced dry season would thus be a typical case of predictably high relative groundwater use.

### A global perspective on groundwater use by plants

A recent multi-site analysis^25^ suggested that groundwater recharge and plant transpiration are supplied by two separate, non-mixed subsurface water pools. This further suggested that plants may only tap water from a single soil-water pool that is not connected to or ever mixes with the groundwater^28^. Using the water isotopic (δ^2^H and δ^18^O) offset relative to the precipitation signature of each water pool (plant xylem water, soil water, groundwater, and/or stream water), Evaristo and colleagues^25^ found that the more highly evaporated (larger offsets) soil and plant xylem waters were statistically distinct from the less evaporated (smaller offsets) stream water and groundwater at 80% of the sites. This approach distinguished between only two belowground water pools (soil and groundwater aquifers) and the different origins of the water that is found in them. From this the authors generalized the ecohydrological patterns previously described for particular sites^23, 27^. We reviewed five studies that were also included in the meta-analysis of Evaristo and colleagues^25^. Importantly, a closer look at the data reveals that even if the water source(s) used by plants (soils in their case) and groundwater isotopic composition are statistically different in general terms, the studies compiled by Evaristo and colleagues^25^ (refs 44–48) still reported considerable (relative) amounts of groundwater uptake by some plants (up to 100% of xylem water, Fig. 4). Therefore, the widespread and consistent isotopic differences found between plant water and groundwater (80% of the sites^25^), may not imply that root water uptake in these sites is not supplied by the same subsurface pools that supply drainage water and groundwater recharge. In fact, the isotopic distinction is usually restricted to the top 30–50 centimeters of the soil^33^, whereas rooting depth expands well below this depth^9, 15, 49^. As a result, the compartmentalization of belowground water in two poorly connected pools may not be a general phenomenon.

Three ecological features of plants can limit the usefulness of plant and soil water isotope data without the adequate high spatial and temporal resolution when testing the two water worlds hypothesis. First, groundwater uptake by plants is likely to be highly seasonal because it occurs when surface soil has been depleted by hot and dry periods^11, 40^ (Figs 1A, 3). Second, coexisting plants may have very different ecohydrological niches^19, 20^, implying that some will take up groundwater, whereas others will rely exclusively on topsoil water. Third, precipitation offset^25^ assumes a unique water source, but plants can use more than one source simultaneously^20, 40^. These three features are overlooked if the isotopic data from one site from different seasons and species are analyzed together, as by Evaristo and colleagues^25^. Therefore, the link between plant transpiration and groundwater pools could be quite strong and occur in more than the 20% of sites that their analytical approach would never reveal. Furthermore, the two water worlds hypothesis assumes that the isotopic distinction is created by the contrasting mobility of belowground water pools. However, isotopic differences are not fully explained by soil water mobility^29^. Alternatively, they may be created by the percolation of precipitation water of successive rain events, soil water evaporation and water storage processes occurring within a well-mixed matrix^5, 33^. Therefore, the ecohydrological separation “that is, poor and incomplete mixing of subsurface water, with one reservoir of water sustaining plant transpiration, and another one contributing to groundwater recharge and streamflow”^25^ is not compatible with the widespread occurrence and large percentages of plant groundwater use reported in this study.

### Concluding remarks

We synthesized data from studies of the use of groundwater using stable-isotope techniques and quantified the relative contributions of those water resources, which were crucial in all biomes represented here, in agreement with previous reports on the global distribution of deep roots^9, 16^. Our results have several implications regarding the representation of the interactions between groundwater dynamics and the biosphere in ESMs. In first place, our synthesis not only confirms that rooting depth usually extends in depth to the water table^11^ but also highlights that the relative contribution of groundwater to transpiration is not affected by the depth to the water table (Fig. 2B). Perhaps more importantly, we show that in sites where the water table or the deep (regional) groundwater pool cannot be accessed by roots (i.e. in slopes or plateaus), the water stored in saprolite or bedrock fractures become water sources of comparable importance to transpiration. This fact urges the consideration of these non-soil (e.g., bedrock-related water pool) in ecohydrological studies^5^ and in ESMs. From those ESMs that include explicit representations of groundwater-ecosystem interactions, most are based on water table levels that can exchange water with the soil profile^12, 50^. According to global estimations of the water table depth, this represents only 7–17% of Earth’s land area^2^. For the remaining lands, bedrock-related water should be incorporated into ESMs. It could be accomplished by considering the first very few meters of bedrock as soil. This can be crucial in areas with shallow rocky soils, and the rooting depth reported by global estimations can be indicative of the depth to consider^17^. In summary, all plant types studied here partly rely on these sources, so groundwater pools should also be considered when studying the plant-water relations of shrublands or grasslands. Insufficient data from boreal and polar regions, however, were available, and few were available from tropical rainforests. Further research on plant-water sources in these regions would thus help our understanding of the global patterns of groundwater uptake and may substantially improve the biosphere-atmosphere models by a realistic representation of this important component of the water cycle.

## Methods

### Data collection

We searched the literature in Google Scholar and the Web of Science for the keywords “water uptake”, “isotopes”, and “mixing model” (last updated 15 June 2015). We assessed all returned studies and selected those that fit the following criteria: (i) used stable isotopes to study plant-water sources, (ii) estimated the relative contribution of groundwater (as defined in reference^1^) to xylem water using mixing models, and (iii) provided estimates of proportional groundwater uptake at the species level. We excluded studies that did not include or explicitly quantify groundwater as a potential source, so we only included studies that directly tested the possibility of roots reaching the groundwater. We also excluded studies that did not account for any belowground water source other than groundwater to obtain the most precise estimate of the relative contribution of groundwater. Thirty-five studies satisfied the selection criteria (Table S1), and 71 species were represented, including herbaceous plants (N = 5), shrubs (N = 17), and trees (N = 50) (one species had both shrub and tree forms). Data were extracted directly from the tables when possible; we used PlotDigitizer 2.6.6 to extract data from figures. The contribution of groundwater to xylem water of each species from each field campaign of each study was recorded as a single datum (N = 545). Each datapoint was then complemented with categorical information for campaign date, rainfall season (dry or wet), and astronomic season (winter, spring, summer, or autumn). Rainfall season was recorded following the categorization made by the original authors of the studies compiled. In those sites with evenly distributed rainfall across all seasons, all data points were considered to belong to wet season. We also defined four categories of landscape position: (i) riparian, plants close to a stream, pond, or oasis; (ii) dunes, plants on sandy dunes either in coastal areas or deserts; (iii) plains, plants in predominantly flat and basal areas but not close to a stream; and (iv) slopes, plants on steep terrain such as hill slopes, hilltops, or mountain plateaus. The data set also included continuous climatic variables from WorldClim 1.4^51^. We downloaded 19 bioclimatic variables using the raster package in R^52^. These variables were derived from the monthly temperatures and rainfall. Finally, we recorded the depth to the groundwater source and the matrix type (bedrock or saturated soil).

### Potential effects of mixing-models on plant water source estimation

In order to quantify the relative contribution of each plant water source, end-member mixing analysis (EMMA) using one or both water isotopes^53^ have been applied to determine the proportional use of each water source. These models assume a complete mixing inside the plant of the contributing water sources and apply a simple equation with the mean value of the isotopic compositions of water sources and xylem water. However, EMMA could only discern among n water sources and required a number of isotopic compositions of n + 1^54^. These simple linear mixing (SLM) models improved substantially in the early 2000s, culminating in the development of IsoSource^55^, a simple program that has subsequently been widely used IsoSource is able to calculate the contributions of more than n + 1 water sources. Its output is rather a range of relative contributions to plant water (see Fig. S2). New isotope mixing models have been developed more recently (SIAR/MixSIAR^56^ and MixSIR^57^) within a Bayesian framework that provide statistical uncertainties associated with the estimates of source contribution and an optimal solution rather than range of feasible solutions^35^. The proportions of each water source has a prior Dirichlet distribution and the posterior distribution is obtained by fitting a linear model to data using a Metropolis-Hasting Markov Chain Monte Carlo algorithm^35, 56^. From the methods described above, a recent inter-comparison review has shown that Bayesian mixing models show the best performance if sampling of soil profile reflects the isotopic gradient in depth^35^. In addition to SLM models, process-based mixing (PBM) models^58^ allow the incorporation of prior information about the system (rooting depth, profiles of root density or soil moisture), and represent a promising avenue to improve the precision of plant-sourcing studies.

We recorded the number of potential sources considered and the number of isotopes used in the mixing models. In addition, IsoSource (the most commonly used mixing model) provides a range of possible solutions, so we recorded these ranges when available to assess potential biases in the estimates of the relative importance of groundwater uptake. The potential biases associated with the application of mixing models were assessed using the same framework of statistical modelling as for the groundwater-use data. We assessed the potential effect of the number of sources other than groundwater used in the mixing model by running a GLMM with relative groundwater uptake as a dependent variable and the number of sources as an explanatory variable, and again, study as a random factor. We also assessed the difference in precision between the dual- and single-isotope approaches for all studies using the IsoSource mixing model and reporting a range of feasible solutions and determined if these potential differences affected the estimation of groundwater use. The mixed models were again run using the MCMCglmm package in R, with study as a random factor.

We found that the number of sources had a significant effect on the isotope mixing models, in which models with fewer potential sources had higher relative contributions of groundwater. This in agreement with a recent study showing the need of a thorough sampling of the soil isotopic gradients^35^. There is increasing isotopic similarity of the water in deep unsaturated (i.e. saprolite water) and saturated layers^5^, both of which originate from rainwater in cold and wet seasons^23^. If this water from unsaturated but deep soil layers is not included in the mixing models, its relative contribution can be confused with that from groundwater, thereby overestimating the importance of the latter^30^. Studies of deep water sources below the soil should thus sample the entire profile overlaying the water table or bedrock, including the fractured rock where possible^5^. The number of isotopes used is another important aspect of the isotope mixing models, as shown by recent studies. Dual-isotope approaches are preferable, because some water pools might differ only in one or the other isotope, so plotting both isotopes is needed to distinguish between them^27^. The studies using the most common isotope mixing model, IsoSource^55^, were more precise when using both isotopes (Fig. S2A). This model returns a range of possible groundwater uses, so using a single isotope will produce wider ranges and thus more uncertainty in the average use. The number of isotopes in our analyses, however, had no effect on the estimated average of relative groundwater uptake and likely had no effect on our results. But studies of plant-water sources are generally enhanced if both water isotopes are used as they generally decrease uncertainty.

### Statistical analyses

Our data set contained 212 records with an average value of relative groundwater uptake for each species, site, and precipitation season (wet or dry) (Table S1)^59–84^. We used these values to create a binary variable: plants with relative contributions of groundwater <0.1 were assigned 0, and plants with relative contribution >0.1 were assigned 1. This format was used to accommodate the output of the mixing models (range of possible solutions, Fig. S1), by which very low contributions can be equal to or higher than the uncertainty of the measure. This data set was used for a hierarchical analysis in which we separately assessed the factors (1) determining the probability of groundwater uptake and (2) explaining the variance of the relative groundwater uptake of those plants that access groundwater pools.

We first fitted a generalized linear mixed model (GLMM) for the probability of groundwater uptake as the dependent variable (binomial distribution, N = 212). Potential explanatory factors were the bioclimatic continuous variables, the edaphic conditions, landscape position, season, and plant type. Interactions among the predictors were also included in an initially saturated model. We included the study as a random factor. The second model analyzed the variance of relative groundwater uptake for contributions >0.1 (N = 155). Isotope mixing-models such as IsoSource provide a range of feasible solutions, which can include zero. For this reason, excluding low mean contributions of groundwater (<10%) ensures the avoidance of false positives. We used the same explanatory, fixed, and random factors as for the first model. In both cases, the best model (the simplest model that maximized the overall fit) for our variables was assessed using the function dredge of the R MuMIn package. This function calculates the goodness of fit of all possible combinations of factors. We used the deviance information criterion (DIC) to select the simplest and most straightforward model from those with a difference <2 DIC points, as the model with the lowest DIC. A separate mixed model was run to test for differences in the relative groundwater uptake between landscape positions. All these mixed models were run with the package MCMCglmm in R.

### Data availability Statement

The authors declare that data used in this study will be available.

## Electronic supplementary material

Supplementary information
