# Supplementary material for: Relative contribution of groundwater to plant transpiration estimated with stable isotopes
Source: Sci Rep. 2017 Sep 5;7:10580. doi: 10.1038/s41598-017-09643-x (PMC5585407; doi:10.1038/s41598-017-09643-x)
Supplement: Supplementary file 1 — Supplementary information [file 41598_2017_9643_MOESM1_ESM.doc]

**Supplementary Information**

Relative contribution of groundwater to plant transpiration estimated with stable isotopes

**Authors**

Adrià Barbeta^1,2,3^*, Josep Peñuelas ^2,3^

Authors’ e-mail addresses:

Adrià Barbeta: [adria.barbeta.margarit@gmail.com](mailto:adria.barbeta.margarit@gmail.com); [a.barbeta@creaf.uab.cat](mailto:a.barbeta@creaf.uab.cat)

Josep Peñuelas: josep.penuelas@uab.cat

**Figure S1. Relative groundwater uptake and the number of sources considered**. Boxplots of the relative groundwater uptake in dry and wet seasons for the number of sources used in the isotope mixing models. Box size represents the interquartile range, the black line is the median, the whiskers indicate variability outside the upper and lower quartiles, and individual points are outliers. The number of sources had a significantly negative effect (P<0.05) on the average groundwater use.

Figure S2. Effect of methodology in studies of plant-water sources. Boxplots of the range of solutions provided by isotope mixing models (A) and the average relative groundwater uptake (B) depending on the number of isotopes used. Box size represents the interquartile range, the black line is the median, the whiskers indicate variability outside the upper and lower quartiles, and individual points are outliers. The asterisk (*) denotes significant differences between groups (P<0.05).

Supplementary Tables

**Table S1.** Summary table of the mean relative groundwater use for each site, species and season.

**Table S2.** Output of the GLMM analyzing the factors determining the probability of plant groundwater utpake. The reference levels from which the posterior mean of the effects have been calculated are: ‘Dry season’ for Precipitation season, ‘Dunes’ for the Landscape position and ‘Herbaceous species’ for Plant type.

**Table S3.** Output of the GLMM analyzing the factors determining the relative contribution of groundwater to plant transpiration. The reference levels from which the posterior mean of the effects have been calculated are: ‘Dry season’ for Precipitation season, ‘Bedrock’ for the Groundwater matrix and ‘Trees’ for Plant type.

Table S1.

| **Study** | **Species** | **Precipitation season** | **Mean relative groundwater use** |
| --- | --- | --- | --- |
| Barbet*a et a*l. 2015 | *Arbutus unedo* | Dry | 0.44 |
| Barbeta *et al.* 2015 | *Arbutus unedo* | Wet | 0.21 |
| Barbeta *et al.* 2015 | *Phillyrea latifolia* | Dry | 0.41 |
| Barbeta *et al.* 2015 | *Phillyrea latifolia* | Wet | 0.24 |
| Barbeta *et al.* 2015 | *Quercus ilex* | Dry | 0.36 |
| Barbeta *et al.* 2015 | *Quercus ilex* | Wet | 0.25 |
| Berr*y et a*l. 2014) | *Abies fraseri* | Wet | 0.19 |
| Berry *et al.* 2014 | *Picea rubens* | Wet | 0.15 |
| Bertran*d et a*l. 2014 | *Alnus incana* | Wet | 0.11 |
| Bertrand *et al.* 2014 | *Pinus sylvestris* | Wet | 0.11 |
| Bertrand *et al.* 2014 | *Populus nigra* | Wet | 0.16 |
| Bertrand *et al.* 2014 | *Prunus avium* | Wet | 0.16 |
| Bertrand *et al.* 2014 | *Salix alba* | Wet | 0.51 |
| Bertrand *et al.* 2014 | *Salix purpurea* | Wet | 0.25 |
| Crame*r et a*l. 1999 (site 1) | *Casuarina glauca* | Dry | 0.75 |
| Cramer *et al.* 1999 (site 1) | *Casuarina glauca* | Wet | 0.45 |
| Cramer *et al.* 1999 (site 2) | *Eucalyptus camaldulensis* | Dry | 0.53 |
| Cramer *et al.* 1999 (site 2) | *Eucalyptus camaldulensis* | Wet | 0.46 |
| Cramer *et al.* 1999 (site 3) | *Casuarina glauca* | Dry | 0.48 |
| Cramer *et al.* 1999 (site 3) | *Casuarina glauca* | Wet | 0.21 |
| Cramer *et al.* 1999 (site 3) | *Eucalyptus camaldulensis* | Dry | 0.21 |
| Cramer *et al.* 1999 (site 3) | *Eucalyptus camaldulensis* | Wet | 0.26 |
| Da*i et a*l. 2014 | *Haloxylon ammodendron* | Dry | 0.90 |
| Dai *et al.* 2014 | *Haloxylon ammodendron* | Wet | 0.00 |
| Dai *et al.* 2014 | *Haloxylon persicum* | Dry | 0.12 |
| Dai *et al.* 2014 | *Haloxylon persicum* | Wet | 0.10 |
| Den*g et a*l. 2014 | *Cyclobalanopsis glauca* | Dry | 0.15 |
| Deng *et al.* 2014 | *Cyclobalanopsis glauca* | Wet | 0.08 |
| Drak*e et a*l. 2011 | *Euclayptus gomphocephala* | Dry | 0.09 |
| Drake *et al.* 2011 | *Euclayptus gomphocephala* | Wet | 0.35 |
| Estrada-Medin*a et a*l. 2013 | *Gymnopodium floribundum* | Dry | 0.75 |
| Estrada-Merina *et al.* 2013 | *Gymnopodium floribundum* | Wet | 0.29 |
| Estrada-Merina *et al.* 2013 | *Piscidia piscipula* | Dry | 0.02 |
| Estrada-Merina *et al.* 2013 | *Piscidia piscipula* | Wet | 0.12 |
| Feikem*a et a*l. 2010 | *Eucalyptus camaldulensis* | Dry | 0.80 |
| Feikema *et al.* 2010 | *Eucalyptus camaldulensis* | Wet | 0.06 |
| Feikema *et al.* 2010 | *Eucalyptus grandis* | Dry | 0.18 |
| Feikema *et al.* 2010 | *Eucalyptus grandis* | Wet | 0.02 |
| G*u et a*l. 2015 | *Cyclobalanopsis glauca* | Dry | 0.13 |
| Gu *et al.* 2015 | *Cyclobalanopsis glauca* | Wet | 0.08 |
| Ha*o et a*l. 2013 | *Populus euphratica* | Dry | 0.84 |
| Hasselquist & Allen 2009 | *Grindelia fraxino-pratensis* | Dry | 0.76 |
| Hasselquist and Allen 2009 | *Grindelia fraxino-pratensis* | Wet | 0.30 |
| Hasselquist and Allen 2009 | *Nitrophila mohavensis* | Dry | 0.11 |
| Hasselquist and Allen 2009 | *Nitrophila mohavensis* | Wet | 0.15 |
| Ji*a et a*l. 2012 | *Caragana intermedia* | Wet | 0.06 |
| Kol*b et a*l. 1997 | *Acer negundo* | Dry | 0.98 |
| Kolb *et al.* 1997 | *Acer negundo* | Wet | 0.77 |
| Lefro*y et a*l. 2001 | *Chamaecytisus proliferus* | Dry | 0.25 |
| Lefroy *et al.* 2001 | *Chamaecytisus proliferus* | Wet | 0.31 |
| L*i et a*l. 2012 | *Artemisia desertorum* | Dry | 0.26 |
| Li *et al.* 2012 | *Artemisia desertorum* | Wet | 0.12 |
| Li *et al.* 2012 | *Caragana korshinskii* | Dry | 0.26 |
| Li *et al.* 2012 | *Caragana korshinskii* | Wet | 0.13 |
| Li *et al.* 2012 | *Pinus tabuliformis* | Dry | 0.37 |
| Li *et al.* 2012 | *Pinus tabuliformis* | Wet | 0.19 |
| Li *et al.* 2012 | *Populus simonii* | Dry | 0.88 |
| Li *et al.* 2012 | *Populus simonii* | Wet | 0.15 |
| Li *et al.* 2012 | *Salix psammophila* | Dry | 0.32 |
| Li *et al.* 2012 | *Salix psammophila* | Wet | 0.16 |
| Li*u et a*l. 2010 | *Gironniera subaequalis* | Dry | 0.39 |
| Liu *et al.* 2010 | *Pometia tomentosa* | Dry | 0.63 |
| Li*u et a*l. 2014 | *Celtis wightii* | Dry | 0.79 |
| Liu *et al.* 2014 | *Cleistanthus sumatranus* | Dry | 0.63 |
| Liu *et al.* 2014 | *Lasiococca comberi* |  | 0.85 |
| Liu *et al.* 2015 Li*u et a*l. 2015 | *Populus euphratica* | Wet | 0.29 |
| Ni*e et a*l. 2010 | *Alchornea trewioides* | Dry | 0.15 |
| Nie *et al.* 2010 | *Alchornea trewioides* | Wet | 0.15 |
| Nie *et al.* 2010 | *Ficus orthoneura* | Wet | 0.96 |
| Nie *et al.* 2010 | *Radermachera sinica* | Dry | 0.93 |
| Nie *et al.* 2010 | *Radermachera sinica* | Wet | 0.48 |
| Nie *et al.* 2010 | *Scheffera octophylla* | Wet | 0.87 |
| Nie *et al.* 2010 | *Stercolia euosma* | Wet | 0.88 |
| Ni*e et a*l. 2012 | *Alchornea trewioides* | Dry | 0.13 |
| Nie *et al.* 2012 | *Radermachera sinica* | Dry | 0.47 |
| Pate & Dawson 1999 | *Banksia prionotes* | Dry | 0.91 |
| Pate & Dawson 1999 | *Banksia prionotes* | Wet | 0.53 |
| Querejet*a et a*l. 2007 | *Brosimum alicastrum* | Dry | 0.07 |
| Quejereta *et al.* 2007 | *Cordia dodecandra* | Dry | 0.59 |
| Querejeta *et al.* 2007 | *Enterolobium cyclocarpum* | Dry | 0.09 |
| Querejeta *et al.* 2007 | *Ficus cotinifolia* | Dry | 0.87 |
| Querejeta *et al.* 2007 | *Spondia spurpurea* | Dry | 0.77 |
| Querejeta *et al.* 2007 | *Talisia olivaeformis* | Dry | 0.03 |
| Sah*a et a*l. 2015 | *Batis maritima* | Dry | 0.43 |
| Saha *et al.* 2015 | *Batis maritima* | Wet | 0.06 |
| Saha *et al.* 2015 | *Capparis flexuosa* | Dry | 0.38 |
| Saha *et al.* 2015 | *Capparis flexuosa* | Wet | 0.09 |
| Saha *et al.* 2015 | *Chromolae frustrata* | Dry | 0.06 |
| Saha *et al.* 2015 | *Chromolae frustrata* | Wet | 0.04 |
| Saha *et al.* 2015 | *Conocarpus erectus* | Dry | 0.30 |
| Saha *et al.* 2015 | *Conocarpus erectus* | Wet | 0.11 |
| Saha *et al.* 2015 | *Eugenia foetida* | Dry | 0.38 |
| Saha *et al.* 2015 | *Eugenia foetida* | Wet | 0.01 |
| Saha *et al.* 2015 | *Piscidia piscipula* | Dry | 0.33 |
| Saha *et al.* 2015 | *Piscidia piscipula* | Wet | 0.48 |
| Schachtschneider & February 2010 | *Acacia erioloba* | Dry | 0.30 |
| Schachtschneider & February 2010 | *Acacia erioloba* | Wet | 0.03 |
| Schachtschneider & February 2010 | *Faidherbia albida* | Dry | 0.52 |
| Schachtschneider & February 2010 | *Faidherbia albida* | Wet | 0.12 |
| Schachtschneider & February 2010 | *Tamarix usneoides* | Dry | 0.19 |
| S*i et a*l. 2014 | *Populus euphratica* | Wet | 0.24 |
| Snyder & Williams 2000 | *Populus fremontii* | Dry | 1.00 |
| Snyder & Williams 2000 | *Populus fremontii* | Wet | 0.85 |
| Snyder & Williams 2000 | *Prosopis velutina* | Dry | 1.00 |
| Snyder & Williams 2000 | *Prosopis velutina* | Wet | 0.72 |
| Snyder & Williams 2000 | *Salix gooddingii* | Dry | 1.00 |
| Snyder & Williams 2000 | *Salix gooddingii* | Wet | 1.00 |
| Snyder & Williams 2003 | *Prosopis velutina* | Dry | 0.33 |
| Snyder & Williams 2003 | *Prosopis velutina* | Wet | 0.07 |
| Son*g et a*l. 2014 | *Pinus sylvestris* | Dry | 0.29 |
| Song *et al.* 2014 | *Pinus sylvestris* | Wet | 0.15 |
| Thorbur*n et a*l. 1993 | *Eucalyptus camaldulensis* | Dry | 0.79 |
| Thorburn *et al.* 1993 | *Eucalyptus camaldulensis* | Wet | 0.73 |
| Thorburn *et al.* 1993 | *Eucalyptus largiflorens* | Dry | 0.88 |
| Thorburn *et al.* 1993 | *Eucalyptus largiflorens* | Wet | 0.87 |
| We*i et a*l. 2013 | *Avicennia marina* | Dry | 0.35 |
| Wei *et al.* 2013 | *Avicennia marina* | Wet | 0.01 |
| Wei *et al.* 2013 | *Casuarina glauca* | Dry | 0.01 |
| Wei *et al.* 2013 | *Casuarina glauca* | Wet | 0.02 |
| Wei *et al.* 2013 | *Melaleuca quinquinervia* | Dry | 0.00 |
| Wei *et al.* 2013 | *Melaleuca quinquinervia* | Wet | 0.00 |
| W*u et a*l. 2013 | *Nitraria tangutorum* | Dry | 0.47 |
| Wu *et al.* 2013 | *Nitraria tangutorum* | Wet | 0.31 |
| Wu *et al.* 2013 | *Reaumuria songarica* | Dry | 0.00 |
| Wu *et al.* 2013 | *Reaumuria songarica* | Wet | 0.00 |
| Wu *et al.* 2013 | *Tamarix ramosissima* | Dry | 0.79 |
| Wu et al. 2013 | *Tamarix ramosissima* | Wet | 0.36 |
| X*u et a*l. 2011 | *Abies faxonia* | Dry | 0.86 |
| Xu *et al.* 2011 | *Abies faxonia* | Wet | 0.84 |
| Xu *et al.* 2011 | *Bashania fangia* | Dry | 0.53 |
| Xu *et al.* 2011 | *Bashania fangia* | Wet | 0.35 |
| Xu *et al.* 2011 | *Betula utilis* | Dry | 0.63 |
| Xu *et al.* 2011 | *Betula utilis* | Wet | 0.35 |
| Yafe*n et a*l. 2012 | *Pinus sylvestris* | Dry | 0.11 |
| Yafen *et al.* 2012 | *Pinus sylvestris* | Wet | 0.01 |
| Zencic*h et a*l. 2002 | *Banksia attenuata* | Dry | 0.45 |
| Zencich *et al.* 2002 | *Banksia attenuata* | Wet | 0.32 |
| Zencich *et al.* 2002 | *Banksia ilicifolia* | Dry | 0.48 |
| Zencich *et al.* 2002 | *Banksia ilicifolia* | Wet | 0.19 |
| Zencich *et al.* 2002 | *Hibbertia hypericoides* | Dry | 0.13 |
| Zencich *et al.* 2002 | *Hibbertia hypericoides* | Wet | 0.09 |

Table S2.

| Factor | Posterior mean | CI: lower 95% | CI: upper 95% | pMCMC |  |
| --- | --- | --- | --- | --- | --- |
| (Intercept) | 325.28 | 197.12 | 458.05 | <0.001 | *** |
| Precipitation season: Wet | -107.90 | -182.98 | -32.60 | 0.004 | ** |
| Landscape position: Plains | -65.68 | -166.58 | 27.81 | 0.17 |  |
| Landscape positions: Slopes | 63.39 | -63.08 | 186.14 | 0.31 |  |
| Landscape positions: Dunes | 4.76 | -163.44 | 174.28 | 0.97 |  |
| Plant type: Shrubs | -141.12 | -243.11 | -40.71 | 0.004 | ** |
| Plant type: Herbaceous | -20.06 | -205.74 | 161.85 | 0.82 |  |
| Precipitation of the dry season | -0.89 | -1.54 | -0.27 | 0.010 | * |
| Mean temperature of the dry season | -0.21 | -0.61 | 0.16 | 0.26 |  |

| Table S3.  Factor | Posterior mean | CI: lower 95% | CI: upper 95% | pMCMC |  |
| --- | --- | --- | --- | --- | --- |
| (Intercept) | 1.16 | 0.86 | 1.46 | < 0.001 | *** |
| Precipitation season: Wet | -0.19 | -0.26 | -0.10 | < 0.001 | *** |
| Plant type: Shrubs | -0.10 | -0.22 | 0.03 | 0.14 |  |
| Plant type: Herbaceous | -0.20 | -0.41 | 0.03 | 0.07 | (*) |
| Groundwater matrix: Saturated soil | -0.30 | -0.63 | 0.01 | 0.06 | (*) |
| Depth to groundwater | -0.20 | -0.34 | -0.05 | 0.007 | ** |
| Depth to groundwater X Groundwater matrix: Saturated soil | 0.20 | 0.05 | 0.33 | 0.008 | ** |
